# Expanding the clinical phenotype of *IARS2*-related mitochondrial disease

**DOI:** 10.1186/s12881-018-0709-3

**Published:** 2018-11-12

**Authors:** Barbara Vona, Reza Maroofian, Emanuele Bellacchio, Maryam Najafi, Kyle Thompson, Ahmad Alahmad, Langping He, Najmeh Ahangari, Abolfazl Rad, Sima Shahrokhzadeh, Paulina Bahena, Falk Mittag, Frank Traub, Jebrail Movaffagh, Nafise Amiri, Mohammad Doosti, Reza Boostani, Ebrahim Shirzadeh, Thomas Haaf, Daria Diodato, Miriam Schmidts, Robert W. Taylor, Ehsan Ghayoor Karimiani

**Affiliations:** 1 Institute of Human Genetics, Julius Maximilians University Würzburg, Würzburg, Germany; 2 Department of Otorhinolaryngology, Head and Neck Surgery, Tübingen Hearing Research Centre (THRC), Eberhard Karls University Tübingen, 72076 Tübingen, Germany; 3 Genetics and Molecular Cell Sciences Research Centre, St George’s, University of London, Cranmer Terrace, London, SW17 0RE UK; 4 Genetics and Rare Diseases, Research Division, ‘Bambino Gesù’ Children Hospital, Rome, Italy; 5 Genome Research Division, Human Genetics Department, Radboud University Medical Center and Radboud Institute for Molecular Life Sciences, Geert Grooteplein Zuid 10, 6525KL, Nijmegen, The Netherlands; 6 Wellcome Centre for Mitochondrial Research, Institute of Neuroscience, Newcastle University, Newcastle upon Tyne, UK; 7 Faculty of Medicine, Mashhad University of Medical Sciences, Mashhad, Iran; 8 Next Generation Genetic Clinic, Mashhad, Iran; 9 Cellular and Molecular Research Center, Sabzevar University of Medical Sciences, Sabzevar, Iran; 10 Department of Orthopaedic Surgery, University Hospital of Tübingen, Hoppe-Seyler-Strasse 3, 72076 Tübingen, Germany; 11 Targeted Drug Delivery Research Center, Pharmaceutical Technology Institute, University of Medical Sciences, Mashhad, Iran; 12 Department of Neurology, Faculty of Medicine, Mashhad University of Medical Sciences, Mashhad, Iran; 13 Sabzevar University of Medical Sciences, Sabzevar, Iran; 14 Unit of Neuromuscular and Neurodegenerative Disorders, Laboratory of Molecular Medicine, ‘Bambino Gesu’ Children’s Research Hospital, Rome, Italy; 15 Center for Pediatrics and Adolescent Medicine, University Hospital Freiburg, Faculty of Medicine, Mathildenstrasse 1, 79112 Freiburg, Germany

**Keywords:** Adrenal insufficiency, CAGSSS, Cataracts, Growth hormone deficiency, *IARS2*, Sensory neuropathy, Sensorineural hearing loss, Skeletal dysplasia, Type II esophageal achalasia

## Abstract

**Background:**

*IARS2* encodes a mitochondrial isoleucyl-tRNA synthetase, a highly conserved nuclear-encoded enzyme required for the charging of tRNAs with their cognate amino acid for translation. Recently, pathogenic *IARS2* variants have been identified in a number of patients presenting broad clinical phenotypes with autosomal recessive inheritance. These phenotypes range from Leigh and West syndrome to a new syndrome abbreviated CAGSSS that is characterised by cataracts, growth hormone deficiency, sensory neuropathy, sensorineural hearing loss, and skeletal dysplasia, as well as cataract with no additional anomalies.

**Methods:**

Genomic DNA from Iranian probands from two families with consanguineous parental background and overlapping CAGSSS features were subjected to exome sequencing and bioinformatics analysis.

**Results:**

Exome sequencing and data analysis revealed a novel homozygous missense variant (c.2625C > T, p.Pro909Ser, NM_018060.3) within a 14.3 Mb run of homozygosity in proband 1 and a novel homozygous missense variant (c.2282A > G, p.His761Arg) residing in an ~ 8 Mb region of homozygosity in a proband of the second family. Patient-derived fibroblasts from proband 1 showed normal respiratory chain enzyme activity, as well as unchanged oxidative phosphorylation protein subunits and IARS2 levels. Homology modelling of the known and novel amino acid residue substitutions in IARS2 provided insight into the possible consequence of these variants on function and structure of the protein.

**Conclusions:**

This study further expands the phenotypic spectrum of IARS2 pathogenic variants to include two patients (patients 2 and 3) with cataract and skeletal dysplasia and no other features of CAGSSS to the possible presentation of the defects in *IARS2*. Additionally, this study suggests that adult patients with CAGSSS may manifest central adrenal insufficiency and type II esophageal achalasia and proposes that a variable sensorineural hearing loss onset, proportionate short stature, polyneuropathy, and mild dysmorphic features are possible, as seen in patient 1. Our findings support that even though biallelic *IARS2* pathogenic variants can result in a distinctive, clinically recognisable phenotype in humans, it can also show a wide range of clinical presentation from severe pediatric neurological disorders of Leigh and West syndrome to both non-syndromic cataract and cataract accompanied by skeletal dysplasia.

**Electronic supplementary material:**

The online version of this article (10.1186/s12881-018-0709-3) contains supplementary material, which is available to authorized users.

## Background

Aminoacyl-tRNA synthetases (ARSs) are evolutionarily conserved enzymes that catalyze amino acid attachment to their cognate tRNA. This catalytic process, termed tRNA charging, is a prerequisite for the translation of genetic sequences into polypeptide chains [1]. Two distinct translation pathways take place in the cytoplasm and in the mitochondria each requiring a separate set of ARSs for the translation of nuclear and mitochondrial genes respectively; in total, there are 37 members of the ARS gene family. Two groups of 17 ARSs are each associated with cytoplasmic or mitochondrial translation, while three so-called bifunctional proteins act in both cellular locations [2, 3]. Based on their cellular localization and function, nomenclature for ARSs follows a systematic scheme that entails the recognised amino acid, followed by ARS for both cytoplasmic and bifunctional enzymes (i.e. IARS for cytoplasmic isoleucyl-tRNA synthetase), and a “2” is added to distinguish mitochondrial ARSs (i.e. IARS2 for mitochondrial isoleucyl-tRNA synthetase) [2, 3]. Although expression of ARSs is ubiquitous and protein synthesis is expected to be systemically impaired, a number of highly diverse clinical phenotypes can emerge from mutations in genes encoding ARSs that affect a wide range of tissues with particularly high metabolic demands [3].

IARS2 is a nuclear-encoded mitochondrial isoleucyl-tRNA synthetase that is imported from the cytosol into the mitochondria where it catalyzes the attachment of an isoleucine residue to a cognate mt-tRNA^Ile^ [4]. Genetic mutations in *IARS2* (OMIM: 612801) on chromosome 1q41 were first described in an extended French-Canadian family with a syndrome abbreviated CAGSSS that is characterised by cataracts, growth hormone deficiency, **s**ensory neuropathy, **s**ensorineural hearing loss, and **s**keletal dysplasia [5]. A detailed follow-up on two previously published first-cousin probands from a genealogically related French-Canadian family dating back to the nineteenth century was also explored [5, 6]. The three probands in this extended family were homozygous for a pathogenic missense variant (c.2726C > T, p.Pro909Leu) in exon 21 of the 23 exon *IARS2* gene. The proline residue exchange affected a predicted anticodon binding domain. An additional Danish proband presenting CAGSSS features was identified with a homozygous pathogenic variant (c.2620C > A, p.Gly874Arg) also affecting exon 21 [7]. One further proband with compound heterozygous mutations (c.1821G > A, p.Trp607*; c.2122G > A, p.Glu708Lys) was diagnosed with Leigh syndrome and died at the age of 18 months [5]. Furthermore, compound heterozygous variants in *IARS2* (case 6: c.607G > C; p.(Gly203Arg) and c.2575 T > C; p.(Phe859Leu)); family 10: c.2446C > T; p.(Arg816*) and c.2575 T > C; p.(Phe859Leu)) have been found in two probands from China with sporadic pediatric cataract which characterised by far the mildest clinical presentation of IARS2-related disorders [8]. Moreover, recently identified novel compound missense variants in *IARS2* (c.680 T > C; p.(Phe227Ser) and c.2450G > A; p.(Arg817His)) have been reported in one family with two Japanese siblings showing milder symptoms of CAGSSS and West syndrome concomitant with Leigh syndrome [9]. Thus, these reports assert that mutations in *IARS2* are responsible for a phenotypic spectrum of rare autosomal recessive disorders that require further clinical characterisation.

In this study, we analysed whole exome sequencing data in two unrelated consanguineous Iranian patients presenting with clinical features overlapping with CAGSSS. Exome analysis revealed different novel homozygous variants in *IARS2* in both families. Patient 1 disclosed a homozygous c.2725C > T, p.Pro909Ser variant which interestingly affects the same p.Pro909 amino acid residue that was described in the French-Canadian family. In the exome analysis of patient 2, whose clinical features were limited to only cataract and skeletal dysplasia, we identified a novel homozygous c.2282A > G, p.His761Arg variant in a region of homozygosity spanning 8 Mb. Additionally, we present a detailed and comparative clinical assessment of patients with a phenotypic spectrum of IARS2-related disorders and illustrate the predicted impact of the identified patient mutations on the protein structure. Moreover, cultured fibroblasts of patient 1 were analysed for mitochondrial enzymatic activity and protein extracts were immunoblotted to assess key mitochondrial oxidative phosphorylation (OXPHOS) proteins and steady-state IARS2 levels. Our patients, who have mild forms of CAGSSS, highlight the wide spectrum of clinical severity associated with *IARS2* mutations. Furthermore, as proband 1 displays CAGSSS symptoms concomitant with growth hormone deficiency, central adrenal insufficiency, as well as type II esophageal achalasia, we propose that the phenotypic spectrum of CAGSSS-related disorders resulting from *IARS2* variants could include this constellation of features.

## Methods

Informed written consent was obtained from the families prior to their inclusion in the study. This study was performed under the tenets of the Declaration of Helsinki and approved by the Ethics Commission of the University of Würzburg (46/15), as well as the Ethical Commission of Sabzevar University of Medical Sciences (IR.medsab.rec.1395.120). Blood samples were collected from all participants. Genomic DNA was extracted from whole blood using a standard salting out method. DNA samples were quantified using Qubit 2.0 (Life Technologies, Carlsbad, CA, USA).

### Next generation sequencing and bioinformatics analysis

Whole exome capture was performed using an Agilent SureSelect Human All Exon V6 Kit according to manufacturer’s recommendations and paired-end sequenced on HiSeq 4000 and HiSeq 2500 sequencers.

Exome data were processed for analysis using a GATK-based pipeline [10] that used Burrows-Wheeler alignment [11] to the GRCh37/hg19 human genome assembly. Variant filtering was performed using the following parameters: exonic variants with flanking splice site regions were filtered for quality, and frequency-based data filtering employed MAF ≤ 0.001 as defined by the 1000 Genomes Project [12] and EVS (ESP6500) (http://evs.gs.washington.edu/EVS/) [accessed May, 2017] population data. Artifact-prone gene families (*HLA*s, *MAGE*s, *MUC*s, *NBPF*s, *OR*s and *PRAME*s) were further excluded and the analysis focused on missense, stopgain/stoploss, startgain/startloss, splicing and indel variants. Variant prioritization was aided by the tools FATHMM [13], MutationAssessor [14], MutationTaster [15], PolyPhen-2 [16], and SIFT [17]. Additionally, the EVS, gnomAD [18], GME Variome Project [19], Iranome [20], Ensembl Variant Table [21], ClinVar [22], and HGMD (2017.1) [23] were used for variant analysis. Variants occurring with MAF > 0.01 in the Iranome were also excluded. Splice sites were analysed using the Alamut Visual 2.7 (Interactive Biosoftware, Rouen, France) splicing module. Putative pathogenic variant prioritization followed the following criteria: either three out of five pathogenicity prediction tools score the variant as deleterious or disease causing, or identified variants or adjacent variants affect an amino acid already associated with a genetic disorder entered in databases such as ClinVar or HGMD. In both cases, population frequency database variant entries are in accordance with standards for frequencies of rare disease causing alleles [24]. The remaining variants were filtered for known disease causing genes first and we prioritized homozygous variants due to the autosomal recessive inheritance pattern of disease and family consanguinity.

### Variant validation and segregation testing

The *IARS2* (GenBank Reference: NM_018060.3) c.2282A > G and c.2725C > T homozygous variants were subjected to bidirectional Sanger sequencing validation with an ABI 3130xl 16-capillary sequencer (Life Technologies, Carlsbad, CA, USA) for segregation analysis in each respective family. Primer sequences that were designed using Primer3 [25] are available upon request. DNA sequence analysis was performed using Gensearch software (Phenosystems SA, Wallonia, Belgium).

### Western blotting

Human fibroblasts were treated as described previously [26]. Proteins of interest were bound by overnight incubation at 4 °C with antibodies against IARS2 (Sigma Prestige cat# HPA024212), COXI (Abcam cat# ab14705, RRID: AB_2084810), SDHA (Abcam cat# ab14715, RRID: AB_301433), Total OXPHOS Human Antibody Cocktail containing antibodies against ATP5A, UQCRC2, SDHB, COXII and NDUFB8 (Abcam cat# ab110411), followed by HRP-conjugated secondary antibodies (Dako Cytomation) and visualised using ECL-prime (GE Healthcare) and BioRad ChemiDoc MP with Image Lab software.

### Assessment of mitochondrial respiratory chain enzyme activities

The activities of individual respiratory chain complexes and the mitochondrial matrix marker enzyme, citrate synthase, were assessed as previously described [27].

### Homology modelling

Homology modelling of the human mitochondrial isoleucine tRNA ligase (IARS2, NCBI: NP_060530.3) was based on the structure of the isoleucine tRNA ligase from *S. aureus* (PDB 1FFY) as the template. IARS2 was built in the amino acid range 58–1012 (sharing 36% amino acid identity with the bacterial homologue) employing the following procedure. All side chain atoms in the template were removed, amino acids were renamed and renumbered to the corresponding human IARS2 residues according to the pairwise sequence alignment (Additional file 1), and the modified PDB file was parsed to SIDEpro [28] for side chain reconstruction.

The tRNA^Ile^ molecule cocrystallized with isoleucine tRNA ligase from *S. aureus* (PDB 1FFY) and Ile-AMS (an isoleucyl-adenylate analogue) cocrystallized with the isoleucine tRNA ligase from *T. thermophilus* (PDB 1JZQ) were added to the human IARS2 model in same binding poses to this ligase as in the crystal structure complexes. Finally, the side chains of the protein were energy minimised applying the Dreiding force field.

## Results

### Case presentations

#### Patient 1 (family 1)

**Fig. 1:**
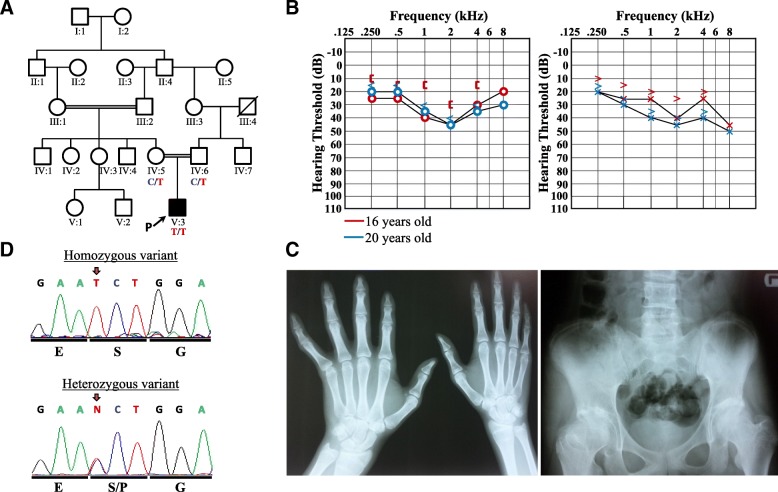
Pedigree of patient 1, pure-tone audiograms, radiological examinations, and sequence electropherogram. **a** The proband’s parents are first-degree cousins. There is no family history of CAGSSS. Genotype results for the c.2725C > T variant are represented under the tested individuals. **b** Pure-tone audiograms from proband 1 at 16 (red) and 20 (blue) years of age reveal stable, sensorineural hearing loss. Right air conduction (circle), unmasked bone conduction (bracket), and masked bone conduction (<) and left air conduction (x) and unmasked bone conduction (>) are shown from left to right, respectively. **c** Radiological images depicting epiphyseal dysplasia of the distal radius and fingers with tapering (left) and right hip with shortened femoral neck due to metaphyseal dysplasia (right). **d** Electropherograms of the homozygous proband (upper panel) and representative heterozygous electrophenogram from a parent (lower panel) showing the nucleotide and amino acid exchange. The variant position is marked with a red arrow

The male proband (Fig. 1a, individual V:3), was born after an uncomplicated pregnancy at 34 weeks gestational age with a 2700 g (25th centile) birth weight and 50 cm (50th centile) length to healthy parents who were first-cousins. At the time of his birth, his father and mother were 29 and 25 years old, respectively. The proband presented with congenital cataracts that required surgery, as well as corneal opacity, bilateral nystagmus, and slight strabismus of the right eye (Table 1, Additional file 2). He was diagnosed with myopia with a visual acuity of 20/40. At birth, he disclosed spastic features and type II achalasia that was confirmed and treated using endoscopy. Throughout early childhood, he experienced delayed motor development; however, this resolved spontaneously later on. No evidence of cognitive performance limitations has been observed.

**Table 1:** Abbreviated clinical summary of patients with pathogenic variants in *IARS2*

	Patient 1 (Present study)	Patient 2 (Present study)	Patient 3 (Present study)	Patient 4 Takezawa et al., 2018 [9]	Patient 5 Takezawa et al., 2018 [9]	Patient 6 Moosa et al., 2017 [7]	Patient 7 (Case 1 in Schwartzentruber et al., 2014 [5]; Jabbour and Harissi-Dagher, 2016 [29]	Patient 8 Case 2 in Schwartzentruber et al., 2014 [5] Patient 1 Liberfarb et al., 1993 [6]	Patient 9 Case 3 in Schwartzentruber et al., 2014 [5] Patient 2 Liberfarb et al., 1993 [6]	Patient 10 Case 4 in Schwartzentruber et al., 2014 [5]
Ethnic descent	Iranian	Iranian	Iranian	Japanese	Japanese	Danish	French-Canadian	French-Canadian	French-Canadian	Scandinavian-Caucasian
Sex	Male	Female	Female	Female	Female	Female	Female	Male	Female	Male
Age at publication	20.6 years	35 years	27 years	8 years	5 years	8 years	33 years	6 years	16.5 years	18 months
Genotype	c.2725C > T p.Pro909Ser	c.2282A > G p.His761Arg	c.2282A > G p.His761Arg	c.680 T > C, p.Phe227Ser; c.2450G > A, p.Arg817His	c.680 T > C, p.Phe227Ser; c.2450G > A, p.Arg817His	c.2620G > A p.Gly874Arg	c.2726C > T p.Pro909Leu	c.2726C > T p.Pro909Leu	c.2726C > T, p.Pro909Leu	c.1821G > A p.Trp607*; c.2122G > A p.Glu708Lys
Ocular evaluation										
Bilateral nystagmus	Yes	Yes	Yes	–	–	Yes	Yes, at 1 month	Yes, at 5 months	Yes, 3 months	–
Cataract	Yes, at birth	Yes, at birth	Yes, at birth	Yes, at birth	–	Yes, at 3 years	Yes, at 17 months; cataract extraction at 22 months	Yes, at 5 months; cataract extraction at 7 months	Yes, 3 months; cataract extracted at 13 months	–
Corneal opacification	Yes	Yes	Yes	–	–	–	Yes, at 5 years, progressive	Yes, at 5 years	Yes, at 16 years 5 months	–
Endocrinology										
Endocrine disturbances	Central adrenal insufficiency, growth hormone deficiency	–	–	–	–	–	Adrenal insufficiency, growth hormone deficiency	–	–	–
Growth hormone replacement therapy	–	–	–	–	–	–	Yes, positive outcome	Yes, positive outcome (cortisol deficiency)	–	–
Hypoglycemic Episodes	–	–	–	–	–	–	Yes	–	Yes	Yes
Auditory evaluation										
Hearing loss	Moderate bilateral sensorineural hearing loss at 13 years of age	–	–	–	–	Bilateral sensorineural hearing loss at 8 years old	Bilateral sensorineural stable hearing loss at 2 years old	Moderate bilateral sensorineural hearing loss at 21 months	–	–
Gastroenterology										
Type II esophageal Achalasia	Yes, from birth	–	–	–	–	–	Yes, 32 years	–	–	–
Musculoskeletal										
Short stature	Yes, proportionate (−3.4 SD)	Yes	Yes	Yes	Yes	Yes, disproportionate (−6 SD),	Yes, disproportionate	Yes	Yes	–
Hip dislocation	–	–	–	–	–	Yes, at birth	Yes, at 2 years	Yes, at birth	Yes, at 18 months	–
Spine abnormalities	Yes, mild scoliosis	–	–	Yes	Yes	Yes, abnormal vertebral bodies	Yes, mild scoliosis	Yes, scoliosis	Yes, scoliosis	
Spondylo-epi-meta- physeal dysplasia	Yes	Yes, disproportional shortening of the first metacarpal reduced bone density	Yes, disproportional shortening of the first metacarpal	–	–	Yes	Yes	Yes	Yes	–
Neurological and Developmental Assessment										
Leigh syndrome features	–	–	–	Yes	Yes	–	–	–	–	Yes
West syndrome	–	–	–	Yes	Yes	–	–	–	–	–
Neurodevelopmental Delay	Yes	–	–	Yes	Yes	Yes	Yes, mild	Yes	Yes	Yes
Current normal Intelligence	Yes	Yes	Yes	–	–	Yes	Yes	–	Yes	?
Peripheral neuropathy	Chronic sensorimotor distal axonal polyneuropathy	–	–	–	–	Yes, pain insensitivity in early childhood	Yes, at 9.5 years	Yes, in early childhood	Yes, at 8 months	–

The proband is presently 20.6 years old. He shows a proportionate short stature (height of 152 cm (− 3.4 SD), a muscle wasted appearance (weight 30 kg, body mass index of 13 kg/m^2^ (− 7.5 SD)) and mild craniofacial dysmorphic features consisting of thick eyebrows, midface retrusion, underdeveloped ala nasi, short philtrum, thin upper lip, vertical crease of the chin, prognatism, and long neck.

A growth hormone deficiency (GHD) was diagnosed, resulting from the combination of severe short stature, delayed bone aged, and very low IGF-1 (− 2 SD) [29]. No growth hormone treatment was available. After repeated measurements, a remarkable decreased serum cortisol level (2.6 μg/dl) and a normal amount of plasmatic adrenocorticotropic hormone (ACTH) in the morning (8 AM) were noted, revealing central adrenal insufficiency. Thyroid-stimulating (TSH), total triiodothyronine (T3), total thyroxine (T4), follicle-stimulating, luteinising, and parathyroid hormones were within reference ranges. An isolated aldosterone measurement showed very high levels at 440 pmol/L (Additional file 2).

Sensorineural hearing loss was diagnosed at 13 years of age (Table 1). Pure-tone audiometry was performed according to best-practice recommendations [30] and revealed a bilateral, stable, mid-to-high frequency moderate hearing loss at ages 16 and 20 years of age (Fig. 1b). Masked bone conduction revealed an air-bone gap at the age of 16 that was later absent. Tympanograms were normal. The patient does not use hearing aids.

Radiological examinations at age 18 years were performed and revealed late manifestations of an underlying spondylo-epi-metaphyseal dysplasia which has been previously described for CAGSSS syndrome. Skeletal abnormalities in patient 1 included mild scoliosis, bilateral pes planus (with arthrodesis of the right ankle joints performed), wrists showing epiphyseal dyplasia of the distal radius, fingers with tapering and a right hip with a shortened femoral neck due to metaphyseal dyplasia with signs of secondary arthrosis of the hip joint (Fig. 1c).

Electrodiagnostic testing of the left limb at 16 years of age showed abnormal results that were consistent with chronic sensorimotor distal axonal polyneuropathy. It revealed extremely low amplitude or absent sensory nerve action potentials and weakening of the upper and lower limbs with a near-normal motor nerve conduction velocity.

Cranial CT and brain MRI were normal (data not shown).

#### Patients 2 and 3 (family 2)

**Fig. 2:**
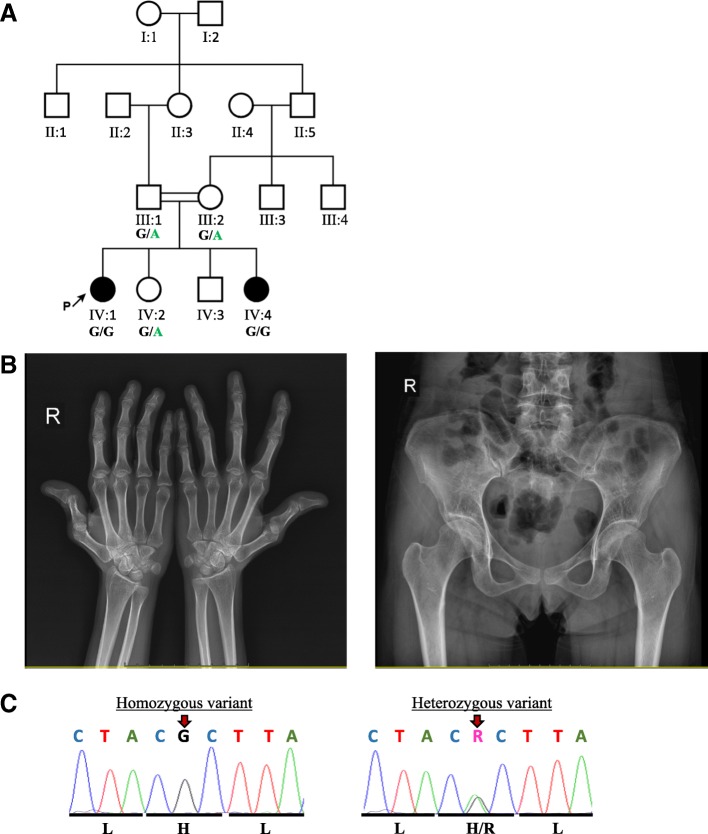
Pedigree of patients 2 and 3, radiological examinations, and sequence electropherogram. **a** The proband’s parents are first-degree cousins. There is no family history of CAGSSS. Genotype results for the c.2282A > G variant are represented under the tested individuals. **b** Radiological images showing that the first metacarpale bone of the right hand (left) projects short and stunted and the plane x-ray the hyperextension of the metacarpophalangeal and the carpophalangeal joints of the thumb is noticeable. The carpal bones show some mild dysplasia and flattening and tapering of the distal phalanges is visible. Some mild dysplasia of the acetabulum is noticeable (right). On both sides a distinct cross over sign is visible. The femoral heads showing some irregular shape with flattened appearance known as pistol grip deformity. The femoral neck seems shortened in comparison to the opposite side. **c** Electropherograms of the homozygous proband (upper panel) and representative heterozygous electropherogram (lower panel) showing the nucleotide and amino acid exchange. The variant position is marked with a red arrow

Family 2, like family 1, is also of Iranian descent with parents being first cousins (Fig. 2a). Two out of four siblings, both females, were diagnosed with cataract at birth. These females are now 35 years old (patient 2) and 27 years old (patient 3). They were both born after an uneventful pregnancy, had a normal birth weight. At birth, no fundus could be identified in patient 2 during ophthalmological investigations and she showed slight strabismus of the right eye. She received a corneal graft later on, which was rejected when she was 30 years-old. She further presented mild craniofacial dysmorphic features consisting of a flat forehead, protruding upper jaw, mildly thickened eyebrows and deep set eyes. Radiological examination showed a mild deformity of the femoral head, small capital femoral epiphyses and hypoplastic, poorly formed acetabular roofs (Fig. 2b). It may be likely that a waddling gait may later develop. Knees show metaphyseal widening and irregularities while hands show brachydactyly (short fingers) and proximal metacarpal rounding. Hearing tests and growth hormone levels were found to be normal.

Her younger sister, patient 3 presented with a similar phenotype of invisible fundus due to the cataract, light strabismus of the right eye and she also received a corneal graft that was rejected when she was 20 years old. Like her older sister, she had normal hearing tests and normal growth hormone levels. She presented with short stature and a similar mild craniofacial dysmorphology as observed in her sister consisting of flattened forehead, protruding upper jaw and mildly thickened eyebrows, as well as deep set eyes (Table 1, Additional file 2).

### Molecular genetic analysis

#### Patient 1 (family 1)

Whole exome sequencing of patient 1 disclosed 26,052 exonic variants. After applying standard quality filtering (22,603 remaining variants), minor allele frequency (MAF) filtering (≤ 0.001) (1563 remaining variants), known artifact prone gene family filtering (1015 remaining variants) and non-synonymous filters, there were 706 variants remaining of which 648 were heterozygous and 58 homozygous. No putative pathogenic heterozygous variants of interest were found. Of the 58 homozygous variants, a single novel homozygous missense c.2725C > T, p.Pro909Ser variant in the gene *IARS2* (GenBank: NM_018060.3, NP_060530.3) was prioritised as a putative pathogenic variant on the basis that this change affects the same residue that was clinically and functionally proven to be causative in a proband with CAGSSS [5] and the patient had clinical features that fit a CAGSSS diagnosis. Further, this variant was found to reside in a large run of homozygosity spanning nearly 14.3 Mb on chr1q41q42.2 that included 102 additional genes (homozygous interval coordinates: rs10779261 to rs2493145; chr1:216,595,306-230,891,248). No variant in any of the other genes within this interval remained after filtering. In silico analysis of synonymous variants in genes within this interval likewise did not reveal any variants predicted to affect splicing. The homozygous *IARS2* variant was confirmed in patient 1 using Sanger sequencing and both parents were found to be heterozygous (Fig. 1d). A variety of in silico pathogenicity prediction tools were used to assess this missense variant (Table 2): MutationTaster predicted the variant to be disease causing, whereas FATHMM, MutationAssessor, PolyPhen-2 and SIFT ranked the consequence of the exchange as tolerated or benign. This apparently novel variant was not deposited in any of the publicly available population frequency databases such as the Exome Variant Server (EVS), gnomAD, Greater Middle Eastern Variome (GME), Iranome, and Ensembl Variant Table (Table 2).

**Table 2:** Pathogenicity and population frequency analysis of *IARS2* variants

*IARS2* Alleles	c.2725C > T, p.Pro909Ser (Present study)	c.2726C > T, p.Pro909Leu	c.2620G > A, p.Gly874Arg	c.2282A > G, p.His761Arg (Present study)	c.680 T > C, p.Phe227Ser	c.2450G > A, p.Arg817His	c.2122G > A, p.Glu708Lys	c.1821G > A, p.Trp607*	c.607G > C p.Gly203Arg	c.2446C > T p.(Arg816*)	c.2575 T > C p.(Phe859Leu)
Phenotypes	CAGSSS	CAGSSS	CAGSSS	Cataract,SD	CAGSSS,Leigh, and West syndrome	CAGSSS,Leigh, and West syndrome	Leigh syndrome	Leigh syndrome	Cataract	Cataract	Cataract
FATHMM	Tolerated (2.93)	Tolerated (2.615)	Tolerated (2.77)	Tolerated (2.57)	Tolerated (0.76)	Tolerated (2.38)	Tolerated (0.96)	No entry	Tolerated (−0.77)	No entry	Tolerated (2.62)
MutationAssessor	Low (1.175)	Medium (2.615)	Medium (2.1)	Medium (2.76)	High (4.34)	High (3.89)	Medium (2.955)	No entry	High (4.265)	No entry	Medium (2.63)
MutationTaster	Disease causing (1)	Disease causing (1)	Disease causing (1)	Disease causing (1)	Disease causing (1)	Disease causing (1)	Disease causing (1)	No entry	Disease causing (1)	No entry	Disease causing (1)
PolyPhen-2	Benign (0.426)	Probably damaging (0.983)	Probably damaging (1.00)	Probably damaging (1.00)	Probably damaging (1.00)	Probably damaging (1.00)	Possibly damaging (0.935)	No entry	Probably damaging (1.00)	No entry	Probably damaging (1.00)
SIFT	Tolerated (0.08)	Deleterious (0.03)	Tolerated (0.41)	Tolerated (0.1)	Deleterious (0)	Tolerated (0.06)	Tolerated (0.06)	No entry	Deleterious (0.01)	No entry	Tolerated (0.4)
Ensembl	Not present	Not present	Not present	Not present	Not present	Allele count: 3; 0 homozygous	Allele count: 6; 1 homozygous	Allele count: 1; 0 homozygous	Not present	Allele count: 1; 0 homozygous	Allele count: 2; 0 homozygous
EVS	Not present	Not present	Allele count: 1; 0 homozygous	Not present	Not present	Allele count: 3; 0 homozygous	Allele count: 11; 0 homozygous	Allele count: 1; 0 homozygous	Not present	Not present	Not present
GME	Not present	Not present	Not present	Not present	Not present	Allele count: 4; 0 homozygous	Allele count: 3; 1 homozygous	Not present	Not present	Not present	Not present
gnomAD	Not present	Not present	Allele count: 6; 0 homozygous	Not present	Not present	Allele count: 4; 0 homozygous	Allele count: 369; 6 homozygous	Allele count: 1; 0 homozygous	Not present	Allele count: 3; 0 homozygous	Allele count:39; 0 homozygous
Iranome	Not present	Not present	Not present	Not present	Not present	Not present	Allele count: 1	Not present	Not present	Not present	Not present

GenBank accession IDs: NM_018060.3, NP_060530.3

#### Patients 2 and 3 (Family 2)

WES was performed on the DNA from patient 2 in the family. On the assumption that the disease follows an autosomal recessive inheritance in the family due to the presence of consanguinity, we prioritised homozygous variants. We further preformed filtering using a minor allele frequency cut off of 0.1% in population databases such as EVS, ExAC, gnomAD, Iranome, and Ensembl given the rare phenotype and excluded non-coding variants except within 10 bp of splice sites as well as synonymous variants. The filtered data narrowed down the variants to a homozygous single nucleotide exchange in exon 18 of *IARS2* currently known monogenic disease-causing genes (c.2282A > G; p.His761Arg) (Fig. 2c) located within an 8 Mb region of homozygosity on chr1q. The variant has not been observed in any public variant database and it is predicted to affect a highly conserved functionally important isoleucyl-tRNA synthetase domain of the protein. The variant was validated by Sanger sequencing, confirming both patient 2 and 3 having the variant in a homozygous state, whereas parents and the only available healthy sibling were heterozygous. No other pathogenic/highly pathogenic variant in currently known monogenic disease-causing genes was identified from the WES data (Mendeliome filtering).

Both variants identified in Families 1 and 2 have been submitted to the Leiden Open Variation Database v.3.0 (LOVD3) with individual IDs 00163754 and 00181201, respectively (https://databases.lovd.nl/shared/genes/IARS2).

### Protein conservation analysis and homology modelling of IARS2 amino acid substitutions

**Fig. 3:**
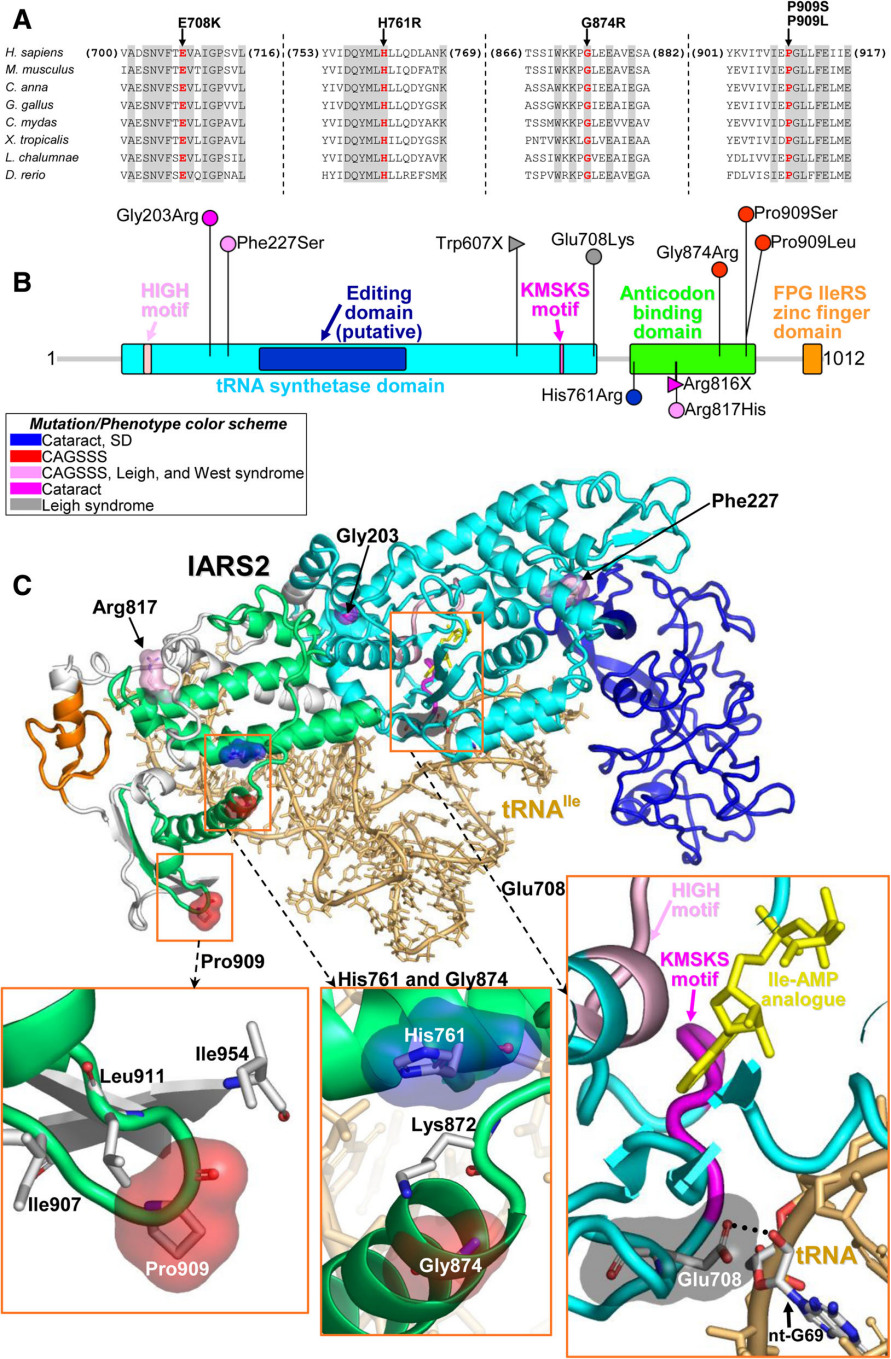
IARS2 scheme, sequence alignment and homology model. **a** Sequence alignment among vertebrates (*H. sapiens*, NP_060530.3; *M. musculus*, NM_198653.2; *C. anna*, XM_008492093.1; *G. gallus*, NM_001006397.1; *C. mydas*, XM_007064764.1; *X. tropicalis*, NM_001127043.1; *L. chalumnae*, XM_005998405.2; *D. rerio*, XM_021467083.1) around the sites of the missense mutations discussed in the text (Glu708Lys, His761Arg, Gly874Arg, Pro909Leu, and Pro909Ser). Residues that are invariant in this group of organisms are shown in gray. **b** Schematic view of IARS2 protein indicating mutations (those reported in this study and the published ones) and colored by phenotype. **c** Homology model of IARS2. The protein ribbon has the same colors that are shown in the functional regions of the protein in panel B. The residues affected by the missense mutations are highlighted by surfaces with the same color scheme as in panel B. The bound cognate tRNA (tRNA^Ile^) is shown as ribbon and sticks in light orange, and the Ile-AMP analogue as yellow sticks

Amino acid conservation analysis and homology modelling of the three previously published and novel IARS2 amino acid substitutions (Glu708Lys, His761Arg, Gly874Glu, Pro909Leu, and Pro909Ser) was performed. For comparative analysis, the Glu708Lys exchange that was reported in compound heterozygosity with a truncating mutation (Trp607*) in the Leigh syndrome proband was included. The Glu708, His761, Gly874, and Pro909 positions are well-conserved to *Danio rerio* (Fig. 3a). IARS2 comprises a tRNA synthetase domain that contains HIGH and KMSKS motifs, an anticodon-binding domain, and an FPG IleRS zinc finger domain (Fig. 3b). The Glu708 residue appears directly involved in the interaction with the tRNA sugar backbone and also in determining IARS2 conformation (Fig. 3c). The Gly874 and Pro909 residues appear to reside in the same anticodon-binding domain.

The His761, Gly874 and Pro909 residues appear to reside in the same anticodon-binding domain. The Gly874Glu substitution affects the N-terminus of an α-helix that interacts with tRNA. Near the latter site of mutation and on another α-helix is where the His761Arg replacement occurs. The Pro909 is part of a loop exposed to the solvent that is not near the active site. It also appears to not be directly involved in tRNA binding (Fig. 3c).

### Functional analysis of cultured fibroblasts

**Fig. 4:**
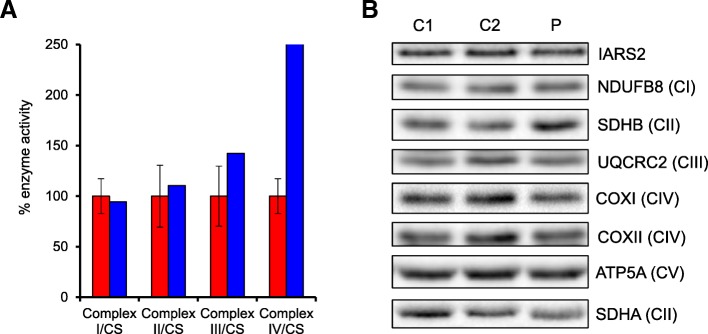
Biochemical and western blot analyses of patient fibroblasts. **a** Activity of mitochondrial respiratory complexes in control (red) and patient (blue) fibroblast samples. Mean enzyme activities normalised to citrate synthase (CS) of control fibroblasts (*n* = 8) are set to 100% and error bars represent standard deviation. **b** Western blots of protein lysate from patient fibroblasts (P) and two age-matched controls (C1 and C2) immunodecorated with antibodies against IARS2, NDUFB8 (CI), UQCRC2 (CIII), COXI (CIV), COXII (CIV) and ATP5A (CV). SDHA and SDHB (CII) were used as loading controls

We next investigated patient fibroblasts of patient 1 to assess whether we could detect a functional consequence of the p.Pro909Ser variant. We detected no obvious defects in any mitochondrial respiratory chain enzyme activities when related to the activity of the mitochondrial matrix enzyme, citrate synthase (Fig. 4a). In agreement with this, the steady-state levels of OXPHOS subunits were unchanged between patient and control fibroblast samples (Fig. 4b). Moreover, steady-state IARS2 protein levels were also unchanged in the patient samples (Fig. 4b).

### Review of clinical presentation of patients with pathogenic variants in *IARS2*

A detailed clinical summary of the seven patients with pathogenic variants in *IARS2* can be found in Additional file 2. A shorter summary presenting the key clinical features observed in the 10 cases known to date can be found in Table 1. The youngest published patient at 8 years of age was from Denmark with a homozygous c.2620G > A, p.Gly874Glu variant [7], whereas the extended French-Canadian family had a segregating c.2726C > T, p.Pro909Leu homozygous variant that involved two previously described patients, patient 1 and patient 2 [6] who were related to an affected individual, case 1 [5]. Also, case 4, with Scandinavian-Caucasian ethnicity had compound heterozygous variants [(c.1821G > A, p.(Trp607*); c.2122G > A, p.(Glu708Lys)] [5]. Of note, case II-1 and II-2 in the Japanese study had segregating compound heterozygous variants [(c.680 T > C; p.[(Phe227Ser)] and c.2450G > A; p. [(Arg817His)] [8]. In the current study, patients in family 1 (patient 1) and family 2 (patient 2 and patient 3) have the c.2725C > T, p.Pro909Ser and c.2282A > G, p.His761Arg homozygous variants, respectively.

### Ocular findings

Ocular findings are amongst the most common features associated with *IARS2* loss of function variants in seven out of ten probands affected by bilateral nystagmus and/or cataracts at birth or within the first three years of life (Table 1). Corneal opacifications have likewise been observed in six out of ten patients. Ocular abnormalities generally appear progressive. Particularly excellent follow-up of the ocular findings of patient 7 in Table 1 (case 1 described by Schwartzentruber and colleagues), included the description of a number of failed corneal grafts and severe eye dryness [31]. Furthermore, this patient had a history of congenital neurotropic keratitis, orbital myopathy and ptosis of the right eye (Table S1). Optical coherence tomography also revealed foveal hypoplasia. Unfortunately, the other cases have not been studied in this detailed manner. However, orbital myopathy and slight strabismus of the right eye are also overlapping phenotypes reported in patient 1.

### Endocrinology

A main feature of CAGSSS is short stature and growth hormone deficiency that was noted in two of ten patients (Table 1). Not all patients may have been investigated in depth and the Danish proband reported normal growth hormone levels at 4 years of age. However, the authors remarked this may be due to her young age [7]. In Table 1, patient 7 had low growth hormone levels at 15 years of age that worsened to a severe deficiency at 22 years of age [5]. Growth hormone replacement therapy was performed in patient 7 and patient 8 with positive outcomes. The presumed central cortisol deficiency of patient 7 would suggest an adrenal insufficiency is present, although this was not directly described [5]. Patient 1 showed a combination of growth hormone deficiency and central adrenal insufficiency, which could be associated with a hypothalamic-pituitary axis dysfunction. Hypoglycemia, which can be linked to central adrenal insufficiency, was noted in patient 7 and patient 9 [32].

### Sensory neuropathy

Half of all patients presented with peripheral neuropathy with a broad age of onset starting as early as 8 months of age (Table 1). A loss of small and medium-sized myelinated fibres, particularly in the hands, was reported in two of these patients. Decreased sensation to pinprick, temperature, and touch in all four extremities was reported in patient 7 [5]. Pain insensitivity was noted in early childhood in another patient [7].

### Sensorineural hearing loss

Four out of ten patients reported to date were found to exhibit sensorineural hearing loss with a broad age of onset ranging from 18 months to 13 years of age (Table 1). Hearing aids were used in only two of these cases (Additional file 2). Previous publications did not show pure-tone audiograms or in-depth audiological testing results. Patient 8 was described with a 60 to 70 dB low-frequency and 50 dB high-frequency hearing loss at the age of 21 months and brainstem auditory evoked potentials corresponding to a 60 dB bilateral sensorineural hearing loss [6]. The most recent audiogram from patient 1 shows gently sloping mid-to-high-frequency moderate sensorineural hearing loss to 45 to 50 dB (Fig. 1c).

### Musculoskeletal alterations

Nine out of ten patients presented with short stature, of which, three exhibited disproportionate short stature and likewise, three cases were diagnosed with spondylo-epi-metaphyseal dysplasia (Table 1). All patients with radiological examinations presented irregular metaphyses and delayed epiphyseal ossification was found in three cases. Seven of ten cases presented with spine abnormalities, of which four presented with scoliosis. Hip dislocation was observed in four cases, joint hypermobility in three cases, and two probands had genu valgum. A muscular wasting appearance was clearly evident in patient 1.

## Discussion

CAGSSS is a rare but highly distinctive syndrome with a unique constellation of features, arising from biallelic mutations in *IARS2.* The patients we described here show a considerable phenotypic overlap to previously described patients. However, not all cases fulfill all features of the CAGSSS acronym. We found significant variability regarding onset of symptoms and some individuals show additional symptoms not included in the acronym, suggesting a wider phenotypic spectrum. With only ten molecularly confirmed cases reported to date, genotype-phenotype correlations are difficult to make with certainty, but it seems possible that there is a predicted milder impact of the p.Pro909Ser variant on IARS2 protein function compared to the other identified alleles (Table 1).

Type II achalasia and adrenal insufficiency were observed in two individuals, patient 1 and patient 7 and interestingly both have a homozygous variant affecting the proline on position 909, p.Pro909Ser in patient 1 and p.Pro909Leu in patient 7 [5]. Due to the low cortisol values in patient 7, we also speculate an adrenal insufficiency that would require further studies. It would be particularly interesting for additional patients with *IARS2* pathogenic variants to be monitored for adrenal insufficiency. The replicated finding of esophageal achalasia suggests an expansion of the CAGSSS phenotypic spectrum. Achalasia, a primary motor disorder of the esophagus, is typically diagnosed in adulthood [33]. Therefore, it is unsurprising that it was not mentioned as a clinical feature in the Danish proband [7]. This may also be an unlikely coincidence that achalasia was also described in a French-Canadian proband [5]. One could speculate that *IARS2* could be one of many genetic factors for this phenotype; however, further reports are required to understand this observation.

In silico analysis suggests the c.2725C > T, p.Pro909Ser variant is likely benign with only one out of five in silico pathogenicity prediction tools ranking this variant as disease causing. Also, two out of five of these tools predicted the novel c.2282A > G, p.His761Arg variant in the current study as disease causing (Table 2). While these tools can be a helpful first insight to the pathogenicity of a variant, no single program provides an error-free prediction result and caution should be used with concluding pathogenicity based exclusively on in silico tools [34]. Based on previous studies, we assessed whether the p.Pro909Ser variant led to any demonstrable effects on OXPHOS function and steady-state IARS2 levels in patient fibroblasts although none were detected. This was somewhat surprising given the earlier report of the patient with the c.2726C > T, p.Pro909Leu variant documented decreased levels of IARS2 protein in patient cells [5]. Despite this, it is well documented that mutation of human mitochondrial ARSs are associated with marked clinical heterogeneity and tissue-specificity, and that cultured skin fibroblasts rarely replicate the functional mitochondrial deficit observed in post-mitotic and clinically-relevant tissues [35–37].

Evolutionary conservation of structurally and functionally important regions is typically a criterion for inferring pathogenicity. Conserved regions have been subjected to negative selection and disease causing variants tend to occur disproportionately in highly conserved amino acids [38]. The homology model of human IARS2 allows the observation that the Glu708Lys variant, a variant associated with Leigh syndrome, affects a glutamic acid directly involved in the binding of mt-tRNA^Ile^ (at the level of nucleotide G69 as inferred from the crystal structure of the complex of tRNA^Ile^ with a bacterial isoleucine tRNA ligase, PDB code 1FFY), and is also very close to the catalytically important KMSKS motif and also nearby the binding site of Ile-AMP (Fig. 3b). The location of Glu708 inside the protein can be reliably assessed from the sequence alignment with the bacterial template (Additional file 1). Thus, it could be expected that the conserved Glu708 might have roles in the correct function of IARS2 and that the Glu708Lys change, which implies a reversal of the electric charge at this site, would somehow influence the enzyme activity. However, the possible clinical importance of the Glu708Lys variant expected from modelling does not appear to be supported by population frequency data, which show a combined eight homozygous (five South Asian individuals, two non-Finnish Europeans, and one Central Asian (GME) individual) and 374 heterozygous carriers from the five population frequency databases used (Table 2) and yielded a combined calculated MAF of 0.00131. In combination with the null Trp607* truncating mutation in the Leigh syndrome patient, [5], we speculate that the Glu708Lys variant alone has moderate severity but may still be functional and provide a viable amount of enzymatic activity but not enough to overcome a null allele. Supported by population frequency data, a homozygous null Trp607* variant is likely to be lethal. We assert that a homozygous Glu708Lys orientation in IARS2 may provide enough enzymatic activity to be viable, and in light of the population frequency data, phenotypically normal. Thus, it remains to be seen whether an association with Leigh syndrome is correct. Phenotypic presentation is also likely absent in the context of variants that mildly disrupt synthetase activity over a certain threshold, since sufficient synthetase activity can be maintained by even low functional activity [39]. Further studies are necessary to understand the role of this variant.

The His761Arg and Gly874Arg amino acid exchanges occur very close to each other (Fig. 3c). The Gly874Arg exchange affects a conserved glycine in the N-terminus of a helix involved in the binding of mt-tRNA^Ile^ (Fig. 3c) and might influence this interaction through conformational changes. As a matter of fact, the Gly874Arg change introduces a large and cationic residue very close to Lys872, thus producing both hindrance and repulsive electric forces promoting conformational changes in the helix. The interactions between the latter helix and the helix bearing His761 might be altered by the replacement of this conserved histidine with an arginine. The Pro909Leu and Pro909Ser variants affect a conserved proline in a solvent exposed loop. Both replacements modify the flexibility of the loop as proline residues provide unique conformational restraints among amino acids. In the case of the Pro909Leu variant, since Pro909 is surrounded by the hydrophobic Ile907, Leu911, and Ile954 residues (Fig. 3c), we foresee that the additional hydrophobic residue introduced by mutation would favour clustering of these hydrophobic residues to minimise their exposure to water. Therefore, the Pro909Leu change is expected to cause more prominent conformational changes compared to the Pro909Ser variant wherein the hydrophilic nature of the loop is preserved by the serine residue. This is consistent with the more severe phenotype associated with the Pro909Leu variant compared to the Pro909Ser variant.

Presently, most of the CAGSSS pathogenic missense variants that have been identified reside in exon 21 (Gly874Arg, Pro909Leu, Pro909Ser) of *IARS2*. It remains unknown whether homozygous variants in this exon tend to be associated with CAGSSS and variants of unknown significance in other affected exons are responsible for Leigh syndrome, a syndrome that is attributed to the death of a patient at 18 months of age that was proposed to be due to compound heterozygous *IARS2* variants (c.1821G > A, p.Trp607* in exon 14 and c.2122G > A, p.Glu708Lys in exon 17) [5]. Japanese siblings who were diagnosed with CAGSSS, Leigh, and West syndrome showed compound heterozygous variants in *IARS2* that affected exons 4 (c.680 T > C, p.Phe227Ser) and 20 (c.2450G > A, p.Arg817His). Patients 2 and 3 presented with ophthalmological and skeletal deficits and had a homozygous variant in exon 18. It can be reasoned that loss-of-function variants result in a more severe phenotype, since tRNA-charging activity would be abolished. This effect is likely independent of the affected exon.

Although no other endocrine disorders have been connected to other patients with mutations in *IARS2*, mitochondrial disease itself represents a high risk for a variety of endocrine diseases. GHD has been related to multiple patients with mitochondrial encephalomyopathy lactic acidosis and stroke-like episodes (MELAS), mtDNA deletions disorders, and nuclear encoded defects [40]. The hypothalamic-pituitary axis dysfunction has been proposed as the underlying pathophysiological mechanism, including chronic ischemia and energy deficiency of the diencephalon, associated with the mitochondrial genetic abnormality of the hypothalamus. This may correlate with our patient, since he shows a co-existing central adrenal insufficiency. Adrenal insufficiency has also been characterised in several patients with other forms of mitochondrial dysfunction, mostly with Kearns-Sayre syndrome, Person syndrome, MELAS, and POLG-related disease [41]. The most accepted pathophysiological mechanism is associated with the high energy demands of endocrine glands; therefore, the impaired mitochondrial ATP production and/or oxidative stress may greatly reduce the ability to secrete hormone or maintain normal feedback [40].

Whilst this manuscript was in peer review, two additional publications described *IARS2* variants in patients. One of the reports described a Japanese family with two siblings who were diagnosed with Leigh syndrome that was concomitant with some of the features of CAGSSS, as well as West syndrome (Table 1 patients 4 and 5) [9]. Both siblings had novel, compound heterozygous *IARS2* [(c.680 T > C; p.[(Phe227Ser)] and c.2450G > A; p. [(Arg817His)] variants in exons 4 and 20, respectively, and exhibited delayed motor development, as well as infantile spasms and abnormal brain MRI diagnostic imaging leading to a diagnosis of Leigh syndrome. One of the two siblings had cataracts and the other sibling had a neonatal hearing screening result requiring follow-up. Both female children were in their first decade of life at the time of publication.

The second publication which characterised by far the mildest clinical presentation characterized to date described two probands from China with sporadic pediatric cataract [8]. Both probands were identified with compound heterozygous variants in *IARS2* (case 6: c.607G > C; p.(Gly203Arg) in exon 4 and c.2575 T > C; p.(Phe859Leu) in exon 21; family 10: c.2446C > T; p.(Arg816*) in exon 20 and c.2575 T > C; p.(Phe859Leu) in exon 21). Both probands shared the Phe859Leu exchange, which affects the anticodon-binding domain and can be reasoned to exhibit only mild/moderate effects. One of the reasons for this hypothesis is that the amino acid exchange conserves hydrophobicity. Another being that the Gly203Arg that is located in the aminoacyl-tRNA synthetase domain and the Arg816* null allele can reasonably be predicted as causing more severe effects on the protein. If the Phe859Leu exchange were to cause a severe protein change, we would expect a severe or even lethal phenotype in the presence of the null allele. Thus, the allelic protein product with the Phe859Leu exchange should still maintain functionality.

## Conclusions

In conclusion, we describe two additional independent families with a total of three affected individuals displaying clinical features overlapping with CAGSSS and novel *IARS2* variants, expanding the clinical and mutational spectrum. Patient 1 also presented with type II esophageal achalasia, as well as growth hormone deficiency and central adrenal insufficiency, which could result from dysfunction of the hypothalamic-pituitary axis. The unusual combination of findings suggest that other endocrine disorders in patients with an *IARS2-*associated mitochondriopathy could likewise be possible and should be excluded. We propose that these additional phenotypes expand the syndromic constellation of symptoms in adults. Early recognition of *IARS2*-related pathophysiology has potentially important implications in clinical management, seeing that undetected adrenal insufficiency could lead to a life threatening crisis.

## Additional files

Additional file 1: Sequence alignment used for homology modelling of IARS2 protein. (DOCX 16 kb)

Additional file 2: Comprehensive clinical summary of patients with pathogenic variants in IARS2. (DOCX 24 kb)

## Abbreviations

ACTH: Adrenocorticotropic hormone
ARS: Aminoacyl-tRNA synthetase
CAGSSS: Cataracts, growth hormone deficiency, sensory neuropathy, sensorineural hearing loss, and skeletal dysplasia
EVS: Exome Variant Server
GHD: Growth hormone deficiency
GME: Greater Middle Eastern Variome project
IARS: Isoleucyl-tRNA synthetase
MAF: Minor allele frequency
MELAS: Mitochondrial encephalomyopathy lactic acidosis and stroke-like episodes
OXPHOS: Oxidative phosphorylation
T3: Total triiodothyronine
T4: Total thyroxine
TSH: Thyroid-stimulating hormone
