# Supplementary material for: Expanding the clinical phenotype of IARS2-related mitochondrial disease
Source: BMC Med Genet. 2018 Nov 12;19:196. doi: 10.1186/s12881-018-0709-3 (PMC6233262; doi:10.1186/s12881-018-0709-3)
Supplement: Supplementary file 1 — Sequence alignment used for homology modelling of IARS2 protein. (DOCX 16 kb) [file 12881_2018_709_MOESM1_ESM.docx]

Additional file 1. IARS2 supplementary alignment modelling

Pairwise sequence alignment (clustal format) employed for the homology modelling of the human mitochondrial isoleucine tRNA ligase (IARS2, NCBI: NP_060530.3), in the residue interval 58-1012, based on the isoleucine tRNA ligase from S. aureus (PDB 1FFY).

---------------------------------------------------------------------------------------------------------

Color scheme of functional regions:

■ tRNA synthetase domain, residues 87-712 (including the putative editing domain)

■ Editing domain, putative, residues 268-461

■ HIGH motif, residues 116-126

■ KMSKS motif, residues 664-668

■ Anticodon-binding domain, residues 756-921

■ FPG IleRS zinc finger domain, residues 984-1008

■ sites of missense variants (E708K, H761R, G874R, P909S/L)

---------------------------------------------------------------------------------------------------------

IARS2 GRYRDTVLLPQTSFPMKLLGRQQPDTELEIQQKCGFSELYSWQRERKV-KTEFCLHDGPP

1FFY MDYEKTLLMPKTDFPMRG---GLPNKEPQIQEKWDAEDQYHKALEKNKGNETFILHDGPP

*..*:*:*:*.***: *:.* :**:* . .: * *:: : * ******

IARS2 YANGDPHVGHALNKILKDIANRFHMMNGSKIHFVPGWDCHGLPIEIKVLSELGREAQNLS

1FFY YANGNLHMGHALNKILKDFIVRYKTMQGFYAPYVPGWDTHGLPIEQALTKKG-VDRKKMS

****: *:**********: *:: *:* :***** ****** : .: : :::*

IARS2 AMEIRKKARSFAKAAIEKQKSAFIRWGIMADWNNCYYTFDGKYEAKQLRTFYQMYDKGLV

1FFY TAEFREKCKEFALEQIELQKKDFRRLGVRGDFNDPYITLKPEYEAAQIRIFGEMADKGLI

: *:*:*.:.** ** **. * * *: .*:*: * *:. :*** *:* * :* ****:

IARS2 YRSYKPVFWSPSSRTALAEAELEYNPEHVSRSIYVKFPLLKPSPKLASLIDGSSPVSILV

1FFY YKGKKPVYWSPSSESSLAEAEIEYHD-KRSASIYVAFNVKDDKGVVDA------DAKFII

*:. ***:*****.::*****:**: : * **** * : . . : : ..:::

IARS2 WTTQPWTIPANEAVCYMPESKYAVVKCSKSGDLYVLAADKVASVASTLET---TFETIST

1FFY WTTTPWTIPSNVAITVHPELKYGQYNV--NGEKYIIAEALSDAVAEALDWDKASIKLEKE

*** *****:* *: ** **. : .*: *::* :**.:*: ::: .

IARS2 LSGVDLENGTCSHPLIPDKASPLLPANHVTMAKGTGLVHTAPAHGMEDYGVASQHNLPMD

1FFY YTGKELEWVVAQHPFL-DRESLVINGDHVTTDAGTGCVHTAPGHGEDDYIVGQQYELPVI

:* :** ...**:: *: * :: .:*** *** *****.** :** *..*::**:

IARS2 CLVDEDGVFTDVAGPELQNKAVLEEGTDVVIKMLQTAKNLLKEEKLVHSYPYDWRTKKPV

1FFY SPIDDKGVFTEEGG-QFEGMFY-DKANKAVTDLLTEKGALLKLDFITHSYPHDWRTKKPV

. :*:.****: .* :::. ::....* .:* *** : :.****:********

IARS2 VIRASKQWFINITDIKTAAKELLKKVKFIPGSALNGMVEMMDRRPYWCISRQRVWGVPIP

1FFY IFRATPQWFASISKVRQDILDAIENTNFKVNWGKTRIYNMVRDRGEWVISRQRVWGVPLP

::**: *** .*:.:: : :::.:* . . . : :*: * * **********:*

IARS2 VFHHKTKDEYLINSQTTEHIVKLVEQHGSDIWWTLPPEQLLPKEVLSEVGGPDALEYVPG

1FFY VFYAEN-GEIIMTKETVNHVADLFAEHGSNIWFEREAKDLLPEGFT--HPGSPNGTFTKE

**: :. .* ::..:*.:*:..*. :***:**: .::***: . *. :.

IARS2 QDILDIWFDSGTSWSYVL---PGPDQRADLYLEGKDQLGGWFQSSLLTSVAARKRAPYKT

1FFY TDIMDVWFDSGSSHRGVLETRPELSFPADMYLEGSDQYRGWFNSSITTSVATRGVSPYKF

**:*:*****:* ** * . **:****.** ***:**: ****:* :***

IARS2 VIVHGFTLGEKGEKMSKSLGNVIHPDVVVNGGQDQSKEPPYGADVLRWWVADSNVFT**E**VA

1FFY LLSHGFVMDGEGKKMSKSLGNVIVPDQVVKQK---------GADIARLWVSSTDYLADVR

:: ***.:. :*:********** ** **: ***: * **:.:: :::*

IARS2 IGPSVLNAARDDISKLRNTLRFLLGNVADFNPETDSIPVNDMYVIDQYML**H**LLQDLANKI

1FFY ISDEILKQTSDDYRKIRNTLRFMLGNINDFNPDTDSIPESELLEVDRYLLNRLREFTAST

*. .:*: : ** *:******:***: ****:***** .:: :*:*:*: *:::: .

IARS2 TELYKQYDFGKVVRLLRTFYTRELSNFYFSIIKDRLYCEKENDPKRRSCQTALVEILDVI

1FFY INNYENFDYLNIYQEVQNFINVELSNFYLDYGKDILYIEQRDSHIRRSMQTVLYQILVDM

: *:::*: :: : ::.* . ******:. ** ** *:.:. *** **.* :** :

IARS2 VRSFAPILPHLAEEVFQHIPYIKEPKSVFRTGWISTSSIWKKP**G**LEEAVESACAMRDSFL

1FFY TKLLAPILVHTAEEVWSHTPHVKE-ESVHLADMPKVVE--VDQALLDKWRTFMNLRDDVN

.: :**** * ****:.* *::** :**. :. .. . . .* : .: :**..

IARS2 GSIPGKNA-------AEYKVITVIE-**P**GLLFEIIEMLQSEETSSTSQLNELMMASESTLL

1FFY RALETARNEKVIGKSLEAKVTIASNDKFNASEFLTSF--------DALHQLFIVSQVKVV

:: . * ** . : *:: : . *::*::.*: .::

IARS2 AQEPREMTADVIELKGKFLINLEGGDIREESSYKVIVMPTTKEKCPRCWKYTAESS----

1FFY DKLDDQATAYE--------------------HGDIVIEHADGEKCERCWNYSEDLGAVDE

: : ** .::: : *** ***:*: : .

IARS2 -DTLCPRCAEVVSGK

1FFY LTHLCPRCQQVVKSL

***** :**..
