# Supplementary material for: Expanding the clinical phenotype of IARS2-related mitochondrial disease
Source: BMC Med Genet. 2018 Nov 12;19:196. doi: 10.1186/s12881-018-0709-3 (PMC6233262; doi:10.1186/s12881-018-0709-3)
Supplement: Supplementary file 2 — Comprehensive clinical summary of patients with pathogenic variants in IARS2. (DOCX 24 kb) [file 12881_2018_709_MOESM2_ESM.docx]

Additional file 2. Comprehensive clinical summary of patients with pathogenic variants in *IARS2*.

|  | Patient 1 from family 1  (Present study) | Patient 2 from family 2  (Present study) | Patient 3 from family 2  (Present study) | Moosa et al., 2017 [7] | Case 1 Schwartzentruber et al., 2014 [5]; Jabbour and Harissi-Dagher, 2016 [27] | Patient 1 Liberfarb et al., 1993 [6] | Patient 2 Liberfarb et al., 1993 [6] |
| --- | --- | --- | --- | --- | --- | --- | --- |
| Ethnic descent | Iranian | Iranian | Iranian | Danish | French-Canadian | French-Canadian | French-Canadian |
| Sex | Male | Female | Female | Female | Female | Male | Female |
| Age at publication | 20.6 years | 35 years | 27 years | 8 years | 33 years | 6 years | 16.5 years |
| **Ocular evaluation** | | | | | | | |
| Bilateral nystagmus | Yes | Yes | Yes | Yes | Yes, at 1 month | Yes, at 5 months | Yes, 3 months |
| Cataracts | Yes, at birth | Yes at birth | Yes at birth | Yes, at 3 years | Yes, at 17 months; cataract extraction at 22 months | Yes, at 5 months; cataract extraction at 7 months | Yes, 3 months; cataract extracted at 13 months |
| Corneal opacification | Yes | Yes | Yes | No | Yes, at 5 years, progressive | Yes, at 5 years | Yes, at 16 years 5 months |
| Strabismus | Slight strabismus of the right eye | No | Mild strabismus (XT) of the right eye | -- | -- | Yes | -- |
| Other findings | 20/40 vision  Orbital myopathy | VA (bilateral):30 cm CF,  Deep set eyes,  Corneal graft was rejected in left eye at age 30 years,  No visible fundus | VA (right):100 cm CF  VA (left): 20 cm CF,  Deep set eyes,  Corneal graft was rejected in left eye at age 20 years,  No visible fundus | Hyperopia | Foveal hypoplasia  Orbital myopathy  Ptosis, right eye | -- | -- |
| **Endocrinology** | | | | | | | |
| Endocrine disturbances | Central adrenal insufficiency  Growth hormone deficiency |  |  | -- | Adrenal insufficiency  Growth hormone deficiency | -- | -- |
| Aldosterone | Isolated measurement 440 pmol/L |  |  | -- | -- | Low at 4 years 10 months | Low at 4 years |
| Cortisol | Deficiency, 2.6 µg/dl (actual therapy 15 mg oral hydrocortisone) |  |  | -- | Deficiency at 15 years (presumed central origin) | -- | -- |
| Insulin-like growth  factor 1 (IGF-1) | -2 SD |  |  | -- | -- | Normal | -- |
| Puberty | Normal |  |  | -- | Normal | -- | -- |
| Triiodothyroxinine  (T3) | Normal |  |  | -- | ~~--~~ | Normal | -- |
| Thyroid-stimulating  hormone (TSH) | Normal |  |  | -- | Normal | -- | -- |
| Thyroxine (T4) | Normal |  |  | -- | Normal | -- | -- |
| Growth hormone  replacement therapy | -- |  |  | -- | Yes, positive outcome | Yes, positive outcome | -- |
| Hypoglycemic  episodes | -- |  |  | -- | Yes | -- | Yes |
| **Auditory evaluation** | | | | | | | |
| Hearing loss | Moderate bilateral sensorineural hearing loss since 13 years of age | Normal | Normal | Bilateral sensorineural hearing loss since 8 years old | Bilateral sensorineural stable hearing loss at 2 years old | Moderate bilateral sensorineural hearing loss at 18 months | Normal |
| Other | Hearing aids not used |  |  |  | Hearing aids used | Hearing aids used; flat pure tone audiogram | -- |
| **Craniofacial dysmorphic features** | | | | | | | |
| Prominent forehead | No | No | No | Yes | Yes | -- | -- |
| Thick eyebrows | Yes | Yes | Yes | No | Yes | -- | -- |
| Infraorbital creases | No | No | No | Yes | No | -- | -- |
| Flat/depressed nasal  bridge | No | No | No | Yes | Yes | Yes | Yes |
| Small nose | No | No | No | Yes | No | -- | -- |
| Hypoplastic nares | No | No | No | Yes | No | -- | -- |
| Deep naso-labial folds | No | No | No | Yes | No | -- | -- |
| Long philtrum | Yes | No | No | -- | -- | Yes | Yes |
| Small mouth | No | No | No | -- | Yes | -- | -- |
| Thin lips | Yes, thin upper lip | No | No | Yes | No | -- | -- |
| Micrognathia | No | No | No | Yes | No | -- | -- |
| **Gastroenterology** | | | | | | | |
| Type II esophageal  achalasia | Yes, from birth | No | No | No | Yes, 32 years | -- | -- |
| Other findings | -- |  |  | -- | Gastro-esophageal sphincter surgery | -- | -- |
| **Musculoskeletal** | | | | | | | |
| Short stature | Yes, proportionate (-3.4 SD) | Yes | Yes | Yes, disproportionate (-6 SD) | Yes, disproportionate | Yes | Yes |
| Cervical spinal  stenosis/cervical  spine instability | No | No | No | -- | Yes | -- | -- |
| Delayed limb  ossification | Yes | No | No | -- | Yes | -- | -- |
| Delayed epiphyseal  ossification | Yes | No | No | Yes | Yes | -- | -- |
| Delayed distal and  proximal femoral  epiphyses ossification | No | No | No | -- | Yes | -- | -- |
| Genu valgum | No | No | No | Yes | Yes | -- | -- |
| Hip dislocation | No | No | No | Yes, at birth | Yes, at 2 years | Yes, at birth | Yes, at 18 months |
| Hyperextensible joints | No | No | No | Yes | -- | -- | -- |
| Hypotonia | No | No | No | Yes | -- | No | -- |
| Irregular metaphyses | Yes |  |  | Yes | Yes | -- | -- |
| Joint hypermobility | No |  |  | Yes, in early childhood | -- | Yes, at birth | Yes |
| Limb proportions | Proportionate | No | No | Shortened limbs | -- | -- | -- |
| Short thorax | No |  |  | Yes |  | -- | -- |
| Shortened long bones | -- |  |  | Yes | Yes | -- | -- |
| Spine abnormalities | Yes, mild scoliosis | No | No | Yes, abnormal vertebral bodies | Yes, mild scoliosis | Yes, scoliosis | Yes, scoliosis |
| Spondylo-epi-meta-physeal dysplasia | Yes | Yes  Disproportional shortening of the first metacarpal | Yes  Disproportional shortening of the first metacarpal | Yes | Yes | Yes | Yes |
| Other findings | Mild osteoporosis | Lost bone density | Lost bone density | Metaphyseal abnormalities | Proximal interphalangeal joint nodules | -- | Camptodactyly of the left 4^th^ and 5^th^ digits and right 5^th^ digit; right leg was 1.5 cm shorter than the left |
| **Neurological and Developmental Assessment** | | | | | | | |
| Birth weight | 2,700 g (25^th^ centile) |  |  | 3,020 g (25^th^ centile) | 2,720 g (25^th^ centile) | 3,011 g (25^th^ centile) | 2,720 g (25^th^ centile) |
| Birth length | 50 cm (50^th^ centile) |  |  | 46 cm (5^th^ centile) | 46.5 cm (5^th^ to 10^th^ centile) | -- | -- |
| Neurodevelopmental delay | Yes | No | No | Yes | Yes, mild | Yes | Yes |
| Current normal intelligence | Yes | Yes | Yes | Yes | Yes | Delayed | Yes |
| Gestation | Birth at 34 weeks of pregnancy | At term | At term | Breech presentation required caesarian section | Uncomplicated | Uncomplicated | Uncomplicated |
| Head circumference | Normal | Norma | Normal | 37 cm (75^th^ centile) | 90^th^ centile | -- | -- |
| Peripheral neuropathy (pain, temperature, touch) | Chronic sensorimotor distal axonal polyneuropathy | Normal | Normal | Yes, pain insensitivity in early childhood | Yes, at 9.5 years | Yes, in early childhood | Yes, at 8 months |
| Other findings | -- |  |  | Hydrocephaly | -- | Loss of small and medium-sized myelinated fibers involving the hands more than the feet | Loss of small and medium-sized myelinated fibers involving the hands more than the feet |
| **Other** | | | | | | | |
| Blistering upon sun  exposure | -- | -- | -- | Yes | -- | -- | -- |
| Bronchiectasis | Yes | -- | -- | -- | -- | -- | -- |
| Poikiloderma | -- | -- | -- | Yes | -- | -- | -- |
| Slow healing injuries | Yes | No | No | Yes (foot ulcers) | Yes | No | No |
| Telangiectasia | -- | No | No | Yes | No | No | No |

--, no information provided

Abbreviations: CF, counting fingers; SD, standard deviation; VA, visual acuity
